# Ethyl 6-methyl-4-[2-(4,4,5,5-tetra­methyl-1,3,2-dioxaborolan-2-yl)thio­phen-3-yl]-2-thioxo-1,2,3,4-tetra­hydro­pyrimidine-5-carboxyl­ate

**DOI:** 10.1107/S1600536808005965

**Published:** 2008-04-26

**Authors:** Andreas Decken, Matthew T. Zamora, Dominique R. Duguay, Christopher M. Vogels, Stephen A. Westcott

**Affiliations:** a Department of Chemistry, University of New Brunswick, Fredericton, NB, Canada E3B 6E2; b Department of Chemistry, Mount Allison University, 63C York Street, Sackville, NB, Canada E4L 1G8

## Abstract

A new Biginelli compound, C_18_H_25_BN_2_O_4_S_2_, containing a boronate ester group was synthesized from a lithium bromide-catalysed reaction. The compound crystallizes with two independent mol­ecules in the asymmetric unit that differ mainly in the conformation of the ester functionality. The crystal structure is stabilized by inter­molecular N—H⋯O and N—H⋯S hydrogen bonds involving the 3,4-dihydro­pyrimidine-2(1*H*)-thione NH groups as donors and the carbonyl O and thio­phene S atoms as acceptors.

## Related literature

Blacquiere *et al.* (2005 ▶) report on previously studied boronic acid Ugi compounds. Miyaura & Suzuki (1995 ▶) give an excellent review on the Suzuki–Miyaura cross-coupling reaction of aryl halides with organoboron derivatives. Vogels *et al.* (2006 ▶) describe the synthesis and characterization of aryl boronate esters derived from aniline. Yang *et al.* (2003 ▶) highlight recent advances of boron chemistry in medicinal research.
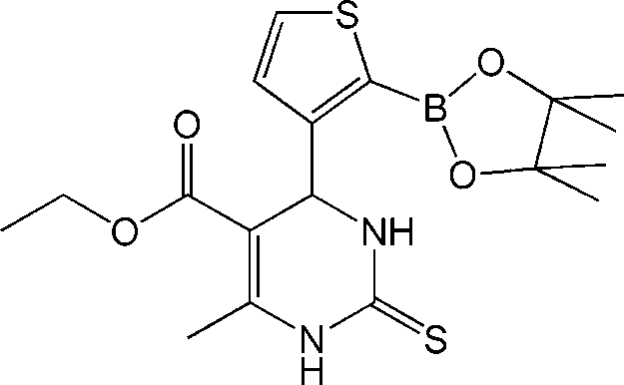


## Experimental

### 

#### Crystal data

C_18_H_25_BN_2_O_4_S_2_
*M* *_r_* = 408.33
Triclinic, 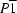

*a* = 11.9274 (17) Å
*b* = 13.5021 (19) Å
*c* = 15.225 (2) Å
α = 112.172 (2)°
β = 93.531 (2)°
γ = 109.706 (2)°
*V* = 2086.9 (5) Å^3^
*Z* = 4
Mo *K*α radiation
μ = 0.28 mm^−1^
*T* = 173 (1) K
0.6 × 0.6 × 0.4 mm

#### Data collection

Bruker SMART1000/P4 diffractometer
Absorption correction: multi-scan (*SADABS*; Sheldrick, 1997 ▶) *T*_min_ = 0.850, *T*_max_ = 0.896
14611 measured reflections
9077 independent reflections
7758 reflections with *I* > 2σ(*I*)
*R*_int_ = 0.017

#### Refinement

*R*[*F* ^2^ > 2σ(*F* ^2^)] = 0.040
*wR*(*F* ^2^) = 0.113
*S* = 1.03
9077 reflections
499 parameters
H-atom parameters constrained
Δρ_max_ = 0.51 e Å^−3^
Δρ_min_ = −0.41 e Å^−3^

### 

Data collection: *SMART* (Bruker, 1999 ▶); cell refinement: *SMART*; data reduction: *SAINT* (Bruker, 2006 ▶); program(s) used to solve structure: *SHELXS97* (Sheldrick, 2008 ▶); program(s) used to refine structure: *SHELXL97* (Sheldrick, 2008 ▶); molecular graphics: *SHELXTL* (Sheldrick, 2008 ▶); software used to prepare material for publication: *SHELXTL*.

## Supplementary Material

Crystal structure: contains datablocks I, global. DOI: 10.1107/S1600536808005965/gk2133sup1.cif

Structure factors: contains datablocks I. DOI: 10.1107/S1600536808005965/gk2133Isup2.hkl

Additional supplementary materials:  crystallographic information; 3D view; checkCIF report

## Figures and Tables

**Table 1:** Hydrogen-bond geometry (Å, °)

*D*—H⋯*A*	*D*—H	H⋯*A*	*D*⋯*A*	*D*—H⋯*A*
N8—H8⋯S3	0.88	2.91	3.7702 (15)	167
N6—H6⋯O49^i^	0.88	2.06	2.8666 (17)	152
N36—H36⋯O19^ii^	0.88	2.14	2.9670 (17)	155
N38—H38⋯S2^iii^	0.88	2.61	3.4817 (14)	170

Symmetry codes: (i) 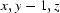; (ii) 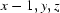; (iii) 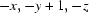.

**Fig. 1.**
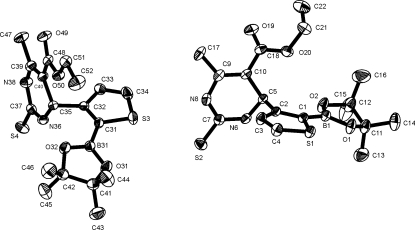
Molecular structure of the title compound with displacement ellipsoids drawn at the 50% probability level. H atoms have been omitted.

## supplementary crystallographic information

### Comment

Compounds containing boronic acids [RB(OH)_2_] or boronate esters [RB(OR')_2_] have found remarkable synthetic utility in Suzuki-Miyaura cross coupling reactions (Miyaura and Suzuki, 1995) over the past decade. Interest in these compounds also arises from their diverse and potent biological activities (Yang *et al.*, 2003). Indeed, we have recently shown that dihydropyrimidinones (Biginelli products) containing boronic acids show significant promise for their ability to inhibit the MCF7 breast cancer cell line (Blacquiere *et al.*, 2005). Biginelli compounds containing thiophenes showed the most promise in this study. Some of the biological properties of boron compounds have been attributed to the ability of the three-coordinate boron atom to form bonds with biomolecules, as well as form hydrogen bonds with the adjacent O atoms of the boronic acid or boronate ester group. We are preparing a family of boron-containing Biginelli products in order to understand the mechanism of action of these compounds in an effort to design more potent candidates.

The title compound crystallizes with two independent molecules per asymmetric unit. In one of the independent molecules the ester group is coplanar with the pyrimidine ring [torsion angles: C40—C48—O50—C51 = 177.11 (13)° and C48—O50—C51—C52 = 179.83 (16)°], the second independent molecule shows rotation of the ethyl group of the ester moiety that displaces the methyl group from the pyrimidine ring plane [torsion angles: C10—C18—O20—C21 = -179.33 (13)° and C18—O20—C21—C22 = -81.2 (2)°]. Conversely, the latter molecule displays coplanar dioxaborolane and thiophene rings [torsion angles: C2—C1—B1—O2 = 6.2 (3)° and S1—C1—B1—O1 = 3.4 (2)°], while the former shows rotation about the inter-ring linkage [torsion angles: C32—C31—B31—O32 = 20.9 (2)° and S3—C31—B31—O31 = 24.7 (3)°]. However, these torsion angles allow for orbital overlap between the boron *p_z_* orbital and the aryl π-electron system. The Bpin skeleton displays similar bond lengths and angles as found in related aniline derivatives (Vogels *et al.*, 2006). Although steric crowding at the boron center is not present, the title compound shows no appreciable intra- or intermolecular Lewis acid-base interactions. However, hydrogen bonding is observed for all N—H groups of the 3,4-dihydropyrimidine-2(1*H*)-thione fragment. Two NH···O bonds are present for H6 and H36 (H6···O49^i ^= 2.06 Å, (i): *x*, *y* - 1, *z* and H36···O19^ii^ = 2.14 Å. (ii): *x* - 1, *y*, *z*) while two very long NH···S bonds are found for H8 and H38 (H8···S3 = 2.91 Å and H38···S2^iii^ = 2.61 Å, (iii): -*x*, -*y* + 1, -*z*).

### Experimental

2-(4,4,5,5-Tetramethyl-1,3,2,-dioxaborolan-3-yl)thiophenecarboxaldehyde (548 mg, 2.30 mmol), ethyl acetoacetate (456 mg, 3.50 mmol) and thiourea (266 mg, 3.49 mmol) were added together with CH_3_CN (15 ml) and a catalytic amount of lithium bromide (40 mg, 0.46 mmol). The reaction was heated at reflux for 60 h. The solvent was reduced to 5 ml and allowed to stand at room temperature. The title compound precipitated as colourless crystals. Yield: 740 mg (79%); m.p. 469 – 471 K.

### Refinement

Hydrogen atoms were included in calculated positions at distances of 0.88 (NH), 0.95 (CH-*sp*^2^), 0.98 (CH_3_), 0.99 (CH_2_), and 1.0 Å (CH-*sp*^3^) from the parent atom and refined using a riding model. *U*_eq_ were 1.5 times of the parent atom for CH_3_ hydrogen atoms and 1.2 times for all remaining hydrogen atoms.

### Crystal data

**Table:**

C_18_H_25_BN_2_O_4_S_2_	*Z* = 4
*M**_r_* = 408.33	*F*_000_ = 864
Triclinic, *P*1	*D*_x_ = 1.300 Mg m^−^^3^
Hall symbol: -P 1	Mo *K*α radiation λ = 0.71073 Å
*a* = 11.9274 (17) Å	Cell parameters from 5490 reflections
*b* = 13.5021 (19) Å	θ = 2.7–28.5º
*c* = 15.225 (2) Å	µ = 0.28 mm^−^^1^
α = 112.172 (2)º	*T* = 173 (1) K
β = 93.531 (2)º	Irregular, colourless
γ = 109.706 (2)º	0.6 × 0.6 × 0.4 mm
*V* = 2086.9 (5) Å^3^	

### Data collection

**Table:**

Bruker SMART1000/P4 diffractometer	7758 reflections with *I* > 2σ(*I*)
Monochromator: graphite	*R*_int_ = 0.017
*T* = 173(1) K	θ_max_ = 27.5º
φ and ω scans	θ_min_ = 1.8º
Absorption correction: multi-scan(SADABS; Sheldrick, 1997)	*h* = −15→15
*T*_min_ = 0.850, *T*_max_ = 0.896	*k* = −16→17
14611 measured reflections	*l* = −19→19
9077 independent reflections	

### Refinement

**Table:**

Refinement on *F*^2^	Secondary atom site location: difference Fourier map
Least-squares matrix: full	Hydrogen site location: inferred from neighbouring sites
*R*[*F*^2^ > 2σ(*F*^2^)] = 0.040	H-atom parameters constrained
*wR*(*F*^2^) = 0.113	*w* = 1/[σ^2^(*F*_o_^2^) + (0.061*P*)^2^ + 0.8428*P*] where *P* = (*F*_o_^2^ + 2*F*_c_^2^)/3
*S* = 1.04	(Δ/σ)_max_ = 0.001
9077 reflections	Δρ_max_ = 0.51 e Å^−^^3^
499 parameters	Δρ_min_ = −0.41 e Å^−^^3^
Primary atom site location: structure-invariant direct methods	Extinction correction: none

### Special details

**Table:**

Geometry. All e.s.d.'s (except the e.s.d. in the dihedral angle between two l.s. planes)are estimated using the full covariance matrix. The cell e.s.d.'s are takeninto account individually in the estimation of e.s.d.'s in distances, anglesand torsion angles; correlations between e.s.d.'s in cell parameters are onlyused when they are defined by crystal symmetry. An approximate (isotropic)treatment of cell e.s.d.'s is used for estimating e.s.d.'s involving l.s. planes.
Refinement. Refinement of *F*^2^ against ALL reflections. The weighted *R*-factor *wR* andgoodness of fit *S* are based on *F*^2^, conventional *R*-factors *R* are basedon *F*, with *F* set to zero for negative *F*^2^. The threshold expression of*F*^2^ > σ(*F*^2^) is used only for calculating *R*-factors(gt) *etc*. and isnot relevant to the choice of reflections for refinement. *R*-factors basedon *F*^2^ are statistically about twice as large as those based on *F*, and *R*-factors based on ALL data will be even larger.

### Fractional atomic coordinates and isotropic or equivalent isotropic displacement parameters (Å^2^)

**Table:**

	*x*	*y*	*z*	*U*_iso_*/*U*_eq_	
B1	0.58518 (16)	0.09209 (15)	0.27239 (12)	0.0268 (3)	
O1	0.61743 (12)	0.02062 (10)	0.30254 (8)	0.0377 (3)	
O2	0.55784 (13)	0.05290 (10)	0.17436 (8)	0.0392 (3)	
C1	0.57648 (13)	0.20455 (12)	0.34609 (10)	0.0241 (3)	
C2	0.53195 (13)	0.27993 (12)	0.33090 (10)	0.0230 (3)	
C3	0.52890 (16)	0.36718 (14)	0.41845 (11)	0.0324 (3)	
H3	0.5002	0.4255	0.4207	0.039*	
C4	0.57164 (17)	0.35812 (14)	0.49885 (11)	0.0346 (4)	
H4	0.5762	0.4091	0.5635	0.042*	
C5	0.48905 (13)	0.27052 (12)	0.23100 (10)	0.0232 (3)	
H5	0.5127	0.2116	0.1819	0.028*	
N6	0.35516 (12)	0.23074 (11)	0.20829 (9)	0.0274 (3)	
H6	0.3125	0.1567	0.1918	0.033*	
C7	0.29331 (14)	0.29623 (13)	0.21037 (10)	0.0271 (3)	
N8	0.36068 (13)	0.40921 (12)	0.22901 (10)	0.0324 (3)	
H8	0.3244	0.4585	0.2442	0.039*	
C9	0.48338 (15)	0.45081 (13)	0.22532 (11)	0.0296 (3)	
C10	0.54748 (14)	0.38534 (13)	0.22401 (10)	0.0262 (3)	
C11	0.59061 (18)	−0.08779 (14)	0.21602 (12)	0.0366 (4)	
C12	0.5912 (2)	−0.04760 (16)	0.13318 (13)	0.0435 (5)	
C13	0.4647 (2)	−0.17160 (18)	0.21154 (18)	0.0577 (6)	
H13A	0.4638	−0.1794	0.2729	0.086*	
H13B	0.4460	−0.2477	0.1580	0.086*	
H13C	0.4035	−0.1415	0.2008	0.086*	
C14	0.6837 (2)	−0.13695 (18)	0.22800 (16)	0.0526 (5)	
H14A	0.7657	−0.0798	0.2377	0.079*	
H14B	0.6682	−0.2079	0.1696	0.079*	
H14C	0.6778	−0.1551	0.2845	0.079*	
C15	0.5030 (3)	−0.1337 (2)	0.03940 (16)	0.0806 (10)	
H15A	0.4195	−0.1507	0.0496	0.121*	
H15B	0.5180	−0.2053	0.0159	0.121*	
H15C	0.5136	−0.1013	−0.0088	0.121*	
C16	0.7206 (3)	−0.0007 (2)	0.1157 (2)	0.0859 (10)	
H16A	0.7208	0.0386	0.0732	0.129*	
H16B	0.7460	−0.0654	0.0850	0.129*	
H16C	0.7775	0.0545	0.1780	0.129*	
C17	0.52927 (18)	0.56795 (15)	0.22327 (14)	0.0409 (4)	
H17A	0.5924	0.6242	0.2819	0.061*	
H17B	0.4616	0.5932	0.2209	0.061*	
H17C	0.5637	0.5629	0.1657	0.061*	
C18	0.67612 (15)	0.42200 (14)	0.21689 (11)	0.0305 (3)	
O19	0.74306 (12)	0.51853 (11)	0.22815 (9)	0.0418 (3)	
O20	0.71312 (11)	0.33367 (11)	0.19778 (9)	0.0371 (3)	
C21	0.83969 (17)	0.35731 (19)	0.18969 (14)	0.0436 (4)	
H21A	0.8652	0.3001	0.2015	0.052*	
H21B	0.8925	0.4356	0.2398	0.052*	
C22	0.8558 (2)	0.3510 (2)	0.09137 (15)	0.0562 (5)	
H22A	0.8012	0.2745	0.0415	0.084*	
H22B	0.9405	0.3628	0.0867	0.084*	
H22C	0.8362	0.4114	0.0814	0.084*	
S1	0.61592 (4)	0.24412 (3)	0.46920 (3)	0.02971 (10)	
S2	0.14012 (4)	0.24790 (4)	0.19037 (3)	0.03629 (11)	
B31	0.02234 (17)	0.68959 (16)	0.39397 (12)	0.0284 (3)	
O31	0.00329 (13)	0.61897 (11)	0.43996 (9)	0.0414 (3)	
O32	−0.01311 (12)	0.77979 (11)	0.43404 (9)	0.0381 (3)	
C31	0.07500 (14)	0.66371 (13)	0.30092 (11)	0.0258 (3)	
C32	0.06028 (13)	0.69246 (12)	0.22483 (10)	0.0238 (3)	
C33	0.11366 (16)	0.64187 (15)	0.14773 (12)	0.0328 (3)	
H33	0.1109	0.6525	0.0895	0.039*	
C34	0.16881 (17)	0.57679 (17)	0.16648 (13)	0.0391 (4)	
H34	0.2099	0.5374	0.1236	0.047*	
C35	−0.00662 (13)	0.76922 (13)	0.22187 (10)	0.0241 (3)	
H35	−0.0143	0.8128	0.2893	0.029*	
N36	−0.13008 (12)	0.69678 (11)	0.15964 (9)	0.0280 (3)	
H36	−0.1828	0.6552	0.1828	0.034*	
C37	−0.17010 (14)	0.68757 (13)	0.07252 (11)	0.0274 (3)	
N38	−0.09181 (13)	0.76047 (12)	0.04064 (9)	0.0315 (3)	
H38	−0.1143	0.7523	−0.0187	0.038*	
C39	0.02087 (15)	0.84658 (14)	0.09561 (11)	0.0287 (3)	
C40	0.06281 (14)	0.85601 (13)	0.18401 (11)	0.0263 (3)	
C41	−0.03602 (17)	0.67207 (16)	0.52899 (12)	0.0356 (4)	
C42	−0.07393 (17)	0.76491 (15)	0.51102 (12)	0.0355 (4)	
C43	−0.1385 (2)	0.5765 (2)	0.53879 (17)	0.0565 (5)	
H43A	−0.2023	0.5341	0.4791	0.085*	
H43B	−0.1728	0.6105	0.5939	0.085*	
H43C	−0.1068	0.5229	0.5497	0.085*	
C44	0.0726 (2)	0.7245 (2)	0.61186 (15)	0.0584 (6)	
H44A	0.1004	0.6640	0.6119	0.088*	
H44B	0.0492	0.7581	0.6735	0.088*	
H44C	0.1386	0.7854	0.6040	0.088*	
C45	−0.2104 (2)	0.7200 (2)	0.46867 (17)	0.0579 (6)	
H45A	−0.2260	0.7746	0.4468	0.087*	
H45B	−0.2564	0.7127	0.5186	0.087*	
H45C	−0.2362	0.6439	0.4134	0.087*	
C46	−0.0335 (2)	0.88144 (19)	0.59638 (15)	0.0570 (6)	
H46A	0.0552	0.9131	0.6192	0.086*	
H46B	−0.0734	0.8726	0.6489	0.086*	
H46C	−0.0561	0.9346	0.5766	0.086*	
C47	0.08465 (18)	0.92231 (16)	0.04776 (13)	0.0403 (4)	
H47A	0.0885	1.0012	0.0839	0.060*	
H47B	0.0395	0.8907	−0.0192	0.060*	
H47C	0.1676	0.9244	0.0474	0.060*	
C48	0.18103 (15)	0.94540 (13)	0.24476 (11)	0.0280 (3)	
O49	0.25664 (11)	1.01361 (11)	0.22269 (9)	0.0409 (3)	
O50	0.19949 (10)	0.94226 (10)	0.33108 (8)	0.0307 (2)	
C51	0.31590 (15)	1.02263 (15)	0.39685 (12)	0.0339 (3)	
H51A	0.3266	1.1037	0.4118	0.041*	
H51B	0.3836	1.0078	0.3673	0.041*	
C52	0.3146 (2)	1.0030 (2)	0.48767 (15)	0.0570 (6)	
H52A	0.2462	1.0165	0.5153	0.085*	
H52B	0.3914	1.0568	0.5350	0.085*	
H52C	0.3054	0.9229	0.4719	0.085*	
S3	0.15505 (4)	0.57455 (4)	0.27683 (3)	0.03606 (11)	
S4	−0.31028 (4)	0.59544 (4)	0.00464 (3)	0.03619 (11)	

### Atomic displacement parameters (Å^2^)

**Table:**

	*U*^11^	*U*^22^	*U*^33^	*U*^12^	*U*^13^	*U*^23^
B1	0.0314 (9)	0.0225 (8)	0.0266 (8)	0.0111 (7)	0.0046 (7)	0.0102 (7)
O1	0.0608 (8)	0.0294 (6)	0.0272 (6)	0.0273 (6)	0.0044 (5)	0.0087 (5)
O2	0.0701 (9)	0.0296 (6)	0.0245 (5)	0.0290 (6)	0.0087 (5)	0.0101 (5)
C1	0.0282 (7)	0.0220 (7)	0.0214 (6)	0.0092 (6)	0.0049 (5)	0.0093 (5)
C2	0.0270 (7)	0.0199 (6)	0.0228 (6)	0.0093 (6)	0.0060 (5)	0.0096 (5)
C3	0.0467 (10)	0.0283 (8)	0.0271 (7)	0.0214 (7)	0.0099 (7)	0.0103 (6)
C4	0.0493 (10)	0.0309 (8)	0.0242 (7)	0.0202 (8)	0.0089 (7)	0.0081 (6)
C5	0.0275 (7)	0.0197 (7)	0.0233 (6)	0.0089 (6)	0.0055 (5)	0.0101 (5)
N6	0.0289 (7)	0.0222 (6)	0.0308 (6)	0.0072 (5)	0.0038 (5)	0.0140 (5)
C7	0.0319 (8)	0.0298 (8)	0.0201 (6)	0.0125 (6)	0.0045 (6)	0.0106 (6)
N8	0.0352 (7)	0.0268 (7)	0.0376 (7)	0.0163 (6)	0.0044 (6)	0.0129 (6)
C9	0.0371 (8)	0.0226 (7)	0.0262 (7)	0.0084 (6)	0.0005 (6)	0.0112 (6)
C10	0.0330 (8)	0.0219 (7)	0.0232 (7)	0.0081 (6)	0.0055 (6)	0.0115 (6)
C11	0.0522 (11)	0.0266 (8)	0.0310 (8)	0.0208 (8)	0.0056 (7)	0.0078 (7)
C12	0.0767 (14)	0.0308 (9)	0.0301 (8)	0.0310 (9)	0.0170 (9)	0.0105 (7)
C13	0.0665 (14)	0.0335 (10)	0.0689 (14)	0.0158 (10)	0.0229 (12)	0.0195 (10)
C14	0.0705 (14)	0.0420 (11)	0.0486 (11)	0.0375 (11)	0.0057 (10)	0.0101 (9)
C15	0.151 (3)	0.0480 (13)	0.0311 (10)	0.0518 (16)	−0.0135 (13)	−0.0019 (9)
C16	0.119 (3)	0.0636 (16)	0.101 (2)	0.0466 (17)	0.083 (2)	0.0432 (16)
C17	0.0500 (11)	0.0251 (8)	0.0476 (10)	0.0113 (8)	−0.0009 (8)	0.0201 (8)
C18	0.0352 (8)	0.0289 (8)	0.0247 (7)	0.0077 (7)	0.0063 (6)	0.0130 (6)
O19	0.0404 (7)	0.0334 (6)	0.0465 (7)	0.0030 (5)	0.0085 (6)	0.0219 (6)
O20	0.0338 (6)	0.0349 (6)	0.0438 (7)	0.0133 (5)	0.0150 (5)	0.0170 (5)
C21	0.0352 (9)	0.0517 (11)	0.0443 (10)	0.0170 (8)	0.0140 (8)	0.0198 (9)
C22	0.0513 (12)	0.0645 (14)	0.0458 (11)	0.0183 (11)	0.0217 (9)	0.0184 (10)
S1	0.0382 (2)	0.0288 (2)	0.02272 (18)	0.01457 (17)	0.00229 (15)	0.01086 (15)
S2	0.0301 (2)	0.0475 (3)	0.0326 (2)	0.01577 (18)	0.00692 (16)	0.01790 (19)
B31	0.0318 (9)	0.0316 (9)	0.0264 (8)	0.0150 (7)	0.0083 (7)	0.0144 (7)
O31	0.0678 (9)	0.0464 (7)	0.0334 (6)	0.0363 (7)	0.0252 (6)	0.0262 (6)
O32	0.0580 (8)	0.0422 (7)	0.0335 (6)	0.0314 (6)	0.0248 (6)	0.0231 (5)
C31	0.0286 (7)	0.0256 (7)	0.0268 (7)	0.0135 (6)	0.0075 (6)	0.0120 (6)
C32	0.0241 (7)	0.0231 (7)	0.0255 (7)	0.0089 (6)	0.0084 (5)	0.0113 (6)
C33	0.0382 (9)	0.0375 (9)	0.0287 (7)	0.0191 (7)	0.0146 (7)	0.0153 (7)
C34	0.0428 (10)	0.0458 (10)	0.0356 (9)	0.0274 (8)	0.0172 (7)	0.0141 (8)
C35	0.0249 (7)	0.0250 (7)	0.0252 (7)	0.0097 (6)	0.0075 (5)	0.0132 (6)
N36	0.0244 (6)	0.0313 (7)	0.0314 (6)	0.0087 (5)	0.0084 (5)	0.0178 (6)
C37	0.0276 (7)	0.0283 (7)	0.0275 (7)	0.0140 (6)	0.0091 (6)	0.0099 (6)
N38	0.0335 (7)	0.0358 (7)	0.0234 (6)	0.0089 (6)	0.0046 (5)	0.0149 (6)
C39	0.0328 (8)	0.0278 (8)	0.0283 (7)	0.0112 (6)	0.0084 (6)	0.0149 (6)
C40	0.0291 (8)	0.0245 (7)	0.0282 (7)	0.0098 (6)	0.0080 (6)	0.0145 (6)
C41	0.0440 (10)	0.0445 (10)	0.0257 (7)	0.0211 (8)	0.0138 (7)	0.0182 (7)
C42	0.0457 (10)	0.0397 (9)	0.0286 (8)	0.0225 (8)	0.0172 (7)	0.0157 (7)
C43	0.0691 (15)	0.0523 (12)	0.0567 (12)	0.0208 (11)	0.0256 (11)	0.0325 (11)
C44	0.0548 (13)	0.0888 (17)	0.0381 (10)	0.0383 (13)	0.0068 (9)	0.0250 (11)
C45	0.0492 (12)	0.0742 (16)	0.0594 (13)	0.0369 (12)	0.0166 (10)	0.0256 (12)
C46	0.0811 (16)	0.0480 (12)	0.0403 (10)	0.0300 (12)	0.0205 (10)	0.0116 (9)
C47	0.0455 (10)	0.0405 (10)	0.0341 (8)	0.0059 (8)	0.0041 (7)	0.0252 (8)
C48	0.0320 (8)	0.0249 (7)	0.0308 (7)	0.0112 (6)	0.0062 (6)	0.0156 (6)
O49	0.0372 (7)	0.0382 (7)	0.0454 (7)	0.0009 (5)	0.0016 (5)	0.0290 (6)
O50	0.0304 (6)	0.0308 (6)	0.0272 (5)	0.0060 (5)	0.0029 (4)	0.0143 (5)
C51	0.0315 (8)	0.0316 (8)	0.0325 (8)	0.0084 (7)	0.0003 (6)	0.0117 (7)
C52	0.0549 (13)	0.0628 (14)	0.0447 (11)	0.0078 (11)	−0.0086 (9)	0.0302 (10)
S3	0.0420 (2)	0.0420 (2)	0.0362 (2)	0.0287 (2)	0.01057 (18)	0.01775 (18)
S4	0.0273 (2)	0.0421 (2)	0.0285 (2)	0.00897 (17)	0.00539 (15)	0.00812 (17)

### Geometric parameters (Å, °)

**Table:**

B1—O2	1.357 (2)	B31—O31	1.352 (2)
B1—O1	1.361 (2)	B31—O32	1.361 (2)
B1—C1	1.553 (2)	B31—C31	1.552 (2)
O1—C11	1.4688 (19)	O31—C41	1.4639 (19)
O2—C12	1.465 (2)	O32—C42	1.4575 (19)
C1—C2	1.378 (2)	C31—C32	1.373 (2)
C1—S1	1.7280 (14)	C31—S3	1.7265 (15)
C2—C3	1.420 (2)	C32—C33	1.425 (2)
C2—C5	1.5185 (19)	C32—C35	1.517 (2)
C3—C4	1.361 (2)	C33—C34	1.357 (2)
C3—H3	0.9500	C33—H33	0.9500
C4—S1	1.7045 (17)	C34—S3	1.7088 (18)
C4—H4	0.9500	C34—H34	0.9500
C5—N6	1.4737 (19)	C35—N36	1.4766 (19)
C5—C10	1.5184 (19)	C35—C40	1.5184 (19)
C5—H5	1.0000	C35—H35	1.0000
N6—C7	1.322 (2)	N36—C37	1.327 (2)
N6—H6	0.8800	N36—H36	0.8800
C7—N8	1.368 (2)	C37—N38	1.369 (2)
C7—S2	1.6836 (16)	C37—S4	1.6808 (16)
N8—C9	1.393 (2)	N38—C39	1.390 (2)
N8—H8	0.8800	N38—H38	0.8800
C9—C10	1.346 (2)	C39—C40	1.350 (2)
C9—C17	1.503 (2)	C39—C47	1.504 (2)
C10—C18	1.470 (2)	C40—C48	1.466 (2)
C11—C14	1.507 (3)	C41—C44	1.509 (3)
C11—C13	1.524 (3)	C41—C43	1.512 (3)
C11—C12	1.549 (2)	C41—C42	1.574 (2)
C12—C15	1.495 (3)	C42—C46	1.505 (3)
C12—C16	1.540 (3)	C42—C45	1.531 (3)
C13—H13A	0.9800	C43—H43A	0.9800
C13—H13B	0.9800	C43—H43B	0.9800
C13—H13C	0.9800	C43—H43C	0.9800
C14—H14A	0.9800	C44—H44A	0.9800
C14—H14B	0.9800	C44—H44B	0.9800
C14—H14C	0.9800	C44—H44C	0.9800
C15—H15A	0.9800	C45—H45A	0.9800
C15—H15B	0.9800	C45—H45B	0.9800
C15—H15C	0.9800	C45—H45C	0.9800
C16—H16A	0.9800	C46—H46A	0.9800
C16—H16B	0.9800	C46—H46B	0.9800
C16—H16C	0.9800	C46—H46C	0.9800
C17—H17A	0.9800	C47—H47A	0.9800
C17—H17B	0.9800	C47—H47B	0.9800
C17—H17C	0.9800	C47—H47C	0.9800
C18—O19	1.216 (2)	C48—O49	1.2187 (19)
C18—O20	1.346 (2)	C48—O50	1.3379 (18)
O20—C21	1.457 (2)	O50—C51	1.4494 (19)
C21—C22	1.495 (3)	C51—C52	1.503 (3)
C21—H21A	0.9900	C51—H51A	0.9900
C21—H21B	0.9900	C51—H51B	0.9900
C22—H22A	0.9800	C52—H52A	0.9800
C22—H22B	0.9800	C52—H52B	0.9800
C22—H22C	0.9800	C52—H52C	0.9800
			
O2—B1—O1	113.78 (14)	O31—B31—O32	114.26 (14)
O2—B1—C1	124.84 (14)	O31—B31—C31	121.32 (15)
O1—B1—C1	121.33 (14)	O32—B31—C31	124.37 (14)
B1—O1—C11	107.00 (12)	B31—O31—C41	108.06 (13)
B1—O2—C12	106.97 (12)	B31—O32—C42	107.96 (12)
C2—C1—B1	130.36 (13)	C32—C31—B31	130.28 (14)
C2—C1—S1	109.91 (11)	C32—C31—S3	110.11 (11)
B1—C1—S1	119.54 (11)	B31—C31—S3	119.35 (11)
C1—C2—C3	113.10 (13)	C31—C32—C33	113.05 (14)
C1—C2—C5	123.78 (12)	C31—C32—C35	123.94 (13)
C3—C2—C5	123.12 (13)	C33—C32—C35	123.01 (13)
C4—C3—C2	112.63 (14)	C34—C33—C32	112.64 (14)
C4—C3—H3	123.7	C34—C33—H33	123.7
C2—C3—H3	123.7	C32—C33—H33	123.7
C3—C4—S1	111.65 (12)	C33—C34—S3	111.55 (12)
C3—C4—H4	124.2	C33—C34—H34	124.2
S1—C4—H4	124.2	S3—C34—H34	124.2
N6—C5—C10	109.92 (12)	N36—C35—C32	109.80 (12)
N6—C5—C2	109.99 (11)	N36—C35—C40	109.87 (12)
C10—C5—C2	111.69 (12)	C32—C35—C40	110.98 (12)
N6—C5—H5	108.4	N36—C35—H35	108.7
C10—C5—H5	108.4	C32—C35—H35	108.7
C2—C5—H5	108.4	C40—C35—H35	108.7
C7—N6—C5	125.95 (13)	C37—N36—C35	127.04 (13)
C7—N6—H6	117.0	C37—N36—H36	116.5
C5—N6—H6	117.0	C35—N36—H36	116.5
N6—C7—N8	116.36 (14)	N36—C37—N38	116.33 (14)
N6—C7—S2	123.93 (12)	N36—C37—S4	123.02 (12)
N8—C7—S2	119.70 (12)	N38—C37—S4	120.63 (12)
C7—N8—C9	123.85 (14)	C37—N38—C39	124.42 (13)
C7—N8—H8	118.1	C37—N38—H38	117.8
C9—N8—H8	118.1	C39—N38—H38	117.8
C10—C9—N8	118.87 (14)	C40—C39—N38	119.54 (14)
C10—C9—C17	127.11 (16)	C40—C39—C47	126.23 (15)
N8—C9—C17	114.02 (15)	N38—C39—C47	114.23 (14)
C9—C10—C18	121.80 (14)	C39—C40—C48	121.59 (14)
C9—C10—C5	120.77 (14)	C39—C40—C35	121.27 (14)
C18—C10—C5	117.42 (13)	C48—C40—C35	117.01 (13)
O1—C11—C14	108.90 (14)	O31—C41—C44	106.68 (15)
O1—C11—C13	106.61 (15)	O31—C41—C43	107.56 (16)
C14—C11—C13	109.29 (17)	C44—C41—C43	110.40 (17)
O1—C11—C12	102.07 (13)	O31—C41—C42	102.79 (12)
C14—C11—C12	116.75 (17)	C44—C41—C42	113.58 (17)
C13—C11—C12	112.48 (17)	C43—C41—C42	115.03 (16)
O2—C12—C15	109.08 (17)	O32—C42—C46	108.64 (16)
O2—C12—C16	106.04 (17)	O32—C42—C45	105.51 (15)
C15—C12—C16	110.6 (2)	C46—C42—C45	109.92 (17)
O2—C12—C11	102.69 (13)	O32—C42—C41	103.19 (12)
C15—C12—C11	116.02 (18)	C46—C42—C41	115.69 (16)
C16—C12—C11	111.7 (2)	C45—C42—C41	113.09 (17)
C11—C13—H13A	109.5	C41—C43—H43A	109.5
C11—C13—H13B	109.5	C41—C43—H43B	109.5
H13A—C13—H13B	109.5	H43A—C43—H43B	109.5
C11—C13—H13C	109.5	C41—C43—H43C	109.5
H13A—C13—H13C	109.5	H43A—C43—H43C	109.5
H13B—C13—H13C	109.5	H43B—C43—H43C	109.5
C11—C14—H14A	109.5	C41—C44—H44A	109.5
C11—C14—H14B	109.5	C41—C44—H44B	109.5
H14A—C14—H14B	109.5	H44A—C44—H44B	109.5
C11—C14—H14C	109.5	C41—C44—H44C	109.5
H14A—C14—H14C	109.5	H44A—C44—H44C	109.5
H14B—C14—H14C	109.5	H44B—C44—H44C	109.5
C12—C15—H15A	109.5	C42—C45—H45A	109.5
C12—C15—H15B	109.5	C42—C45—H45B	109.5
H15A—C15—H15B	109.5	H45A—C45—H45B	109.5
C12—C15—H15C	109.5	C42—C45—H45C	109.5
H15A—C15—H15C	109.5	H45A—C45—H45C	109.5
H15B—C15—H15C	109.5	H45B—C45—H45C	109.5
C12—C16—H16A	109.5	C42—C46—H46A	109.5
C12—C16—H16B	109.5	C42—C46—H46B	109.5
H16A—C16—H16B	109.5	H46A—C46—H46B	109.5
C12—C16—H16C	109.5	C42—C46—H46C	109.5
H16A—C16—H16C	109.5	H46A—C46—H46C	109.5
H16B—C16—H16C	109.5	H46B—C46—H46C	109.5
C9—C17—H17A	109.5	C39—C47—H47A	109.5
C9—C17—H17B	109.5	C39—C47—H47B	109.5
H17A—C17—H17B	109.5	H47A—C47—H47B	109.5
C9—C17—H17C	109.5	C39—C47—H47C	109.5
H17A—C17—H17C	109.5	H47A—C47—H47C	109.5
H17B—C17—H17C	109.5	H47B—C47—H47C	109.5
O19—C18—O20	123.16 (16)	O49—C48—O50	122.17 (14)
O19—C18—C10	126.16 (16)	O49—C48—C40	127.06 (14)
O20—C18—C10	110.66 (13)	O50—C48—C40	110.75 (13)
C18—O20—C21	117.03 (14)	C48—O50—C51	116.55 (12)
O20—C21—C22	111.17 (16)	O50—C51—C52	106.59 (14)
O20—C21—H21A	109.4	O50—C51—H51A	110.4
C22—C21—H21A	109.4	C52—C51—H51A	110.4
O20—C21—H21B	109.4	O50—C51—H51B	110.4
C22—C21—H21B	109.4	C52—C51—H51B	110.4
H21A—C21—H21B	108.0	H51A—C51—H51B	108.6
C21—C22—H22A	109.5	C51—C52—H52A	109.5
C21—C22—H22B	109.5	C51—C52—H52B	109.5
H22A—C22—H22B	109.5	H52A—C52—H52B	109.5
C21—C22—H22C	109.5	C51—C52—H52C	109.5
H22A—C22—H22C	109.5	H52A—C52—H52C	109.5
H22B—C22—H22C	109.5	H52B—C52—H52C	109.5
C4—S1—C1	92.71 (7)	C34—S3—C31	92.65 (8)
			
C2—C1—B1—O2	6.2 (3)	C18—O20—C21—C22	−81.2 (2)
S1—C1—B1—O1	3.4 (2)	C48—O50—C51—C52	179.83 (16)
C32—C31—B31—O32	−24.7 (3)	C10—C18—O20—C21	−179.33 (13)
S3—C31—B31—O31	−20.9 (2)	C40—C48—O50—C51	177.11 (13)

### Hydrogen-bond geometry (Å, °)

**Table:**

*D*—H···*A*	*D*—H	H···*A*	*D*···*A*	*D*—H···*A*
N8—H8···S3	0.88	2.91	3.7702 (15)	167
N6—H6···O49^i^	0.88	2.06	2.8666 (17)	152
N36—H36···O19^ii^	0.88	2.14	2.9670 (17)	155
N38—H38···S2^iii^	0.88	2.61	3.4817 (14)	170

Symmetry codes: (i) *x*, *y*−1, *z*; (ii) *x*−1, *y*, *z*; (iii) −*x*, −*y*+1, −*z*.
